# Supplementary material for: Predicting visual function by interpreting a neuronal wiring diagram
Source: Nature. 2024 Oct 2;634(8032):113–23. doi: 10.1038/s41586-024-07953-5 (PMC11446822; doi:10.1038/s41586-024-07953-5)
Supplement: Supplementary file 6 — CRF and ERF predictions for individual TmY4 and TmY9 cells. Analogous to Supplementary Data 3, but for TmY target types. Shown are the top four monosynaptic pathways, the strongest pathway passing through each of the top ten intermediary types (ranking from Extended Data Fig. 7), and the trisynaptic pathway Tm1–TmY–Dm3–TmY (see the section entitled Prediction of spatial normalization). [file 41586_2024_7953_MOESM6_ESM.zip › DataS4/TmY9q/720575940639801678.pdf]

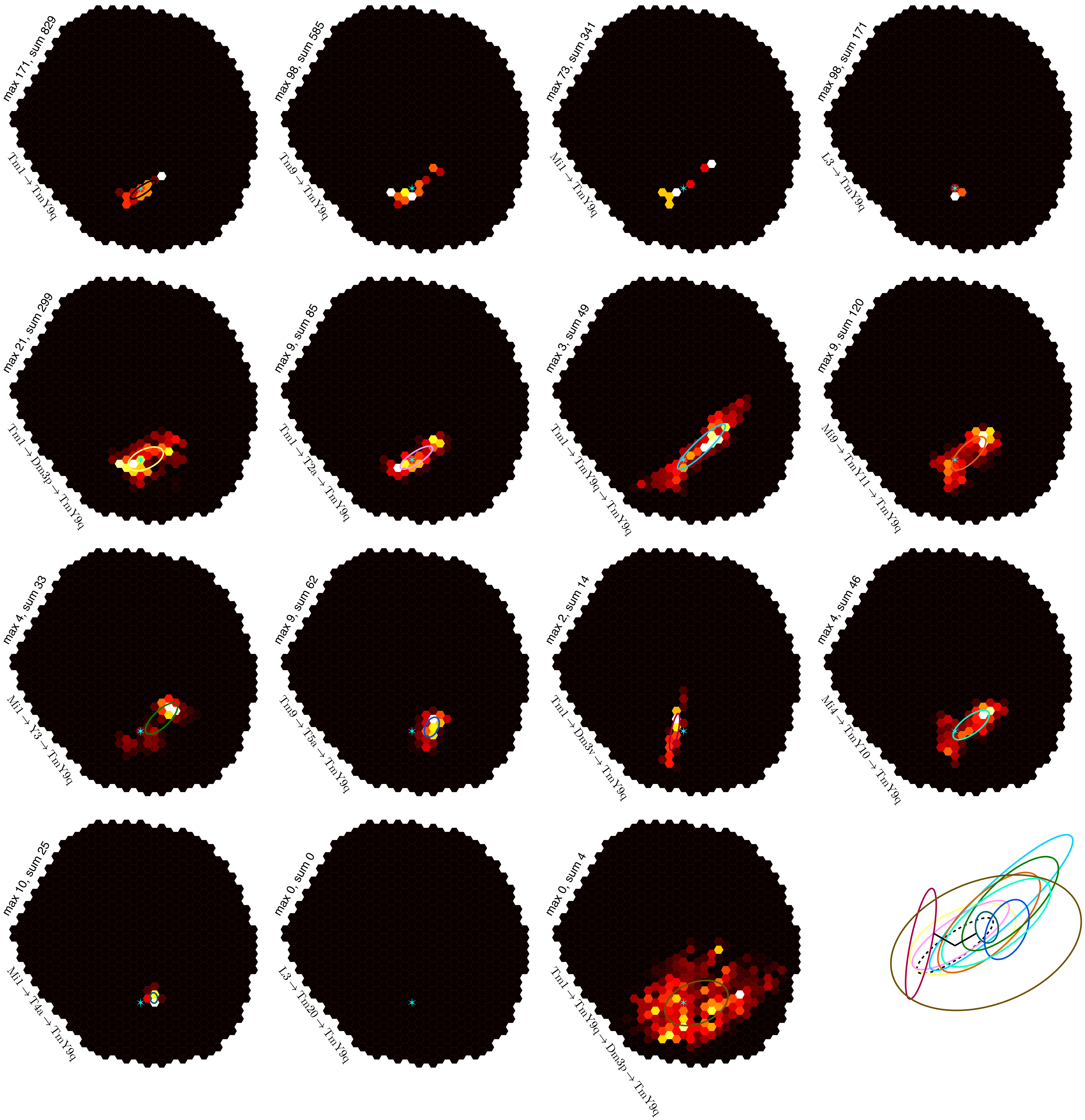
max 171, sum 829
Tm1 → TmY9q
max 98, sum 585
Tm9 → TmY9q
max 73, sum 341
Mi1 → TmY9q
max 98, sum 171
L3 → TmY9q
max 21, sum 299
Tm1 → Dm3p → TmY9q
max 9, sum 85
Tm1 → T2a → TmY9q
max 3, sum 49
Tm1 → TmY9q → TmY9q
max 9, sum 120
Mi9 → TmY11 → TmY9q
max 4, sum 33
Mi1 → Y3 → TmY9q
max 9, sum 62
Tm9 → T5a → TmY9q
max 2, sum 14
Tm1 → Dm3v → TmY9q
max 4, sum 46
Mi4 → TmY10 → TmY9q
max 10, sum 25
Mi1 → T4a → TmY9q
max 0, sum 0
L3 → Tm20 → TmY9q
max 0, sum 4
Tm1 → TmY9q → Dm3p → TmY9q
